# The Dopamine System and Automatization of Movement Sequences: A Review With Relevance for Speech and Stuttering

**DOI:** 10.3389/fnhum.2021.661880

**Published:** 2021-12-02

**Authors:** Per A. Alm

**Affiliations:** Department of Neuroscience, Uppsala University, Uppsala, Sweden

**Keywords:** dopamine, automatization, speech, movement sequences, chunking, basal ganglia, stuttering, Parkinson’s disease

## Abstract

The last decades of research have gradually elucidated the complex functions of the dopamine system in the vertebrate brain. The multiple roles of dopamine in motor function, learning, attention, motivation, and the emotions have been difficult to reconcile. A broad and detailed understanding of the physiology of cerebral dopamine is of importance in understanding a range of human disorders. One of the core functions of dopamine involves the basal ganglia and the learning and execution of automatized sequences of movements. Speech is one of the most complex and highly automatized sequential motor behaviors, though the exact roles that the basal ganglia and dopamine play in speech have been difficult to determine. Stuttering is a speech disorder that has been hypothesized to be related to the functions of the basal ganglia and dopamine. The aim of this review was to provide an overview of the current understanding of the cerebral dopamine system, in particular the mechanisms related to motor learning and the execution of movement sequences. The primary aim was not to review research on speech and stuttering, but to provide a platform of neurophysiological mechanisms, which may be utilized for further research and theoretical development on speech, speech disorders, and other behavioral disorders. Stuttering and speech are discussed here only briefly. The review indicates that a primary mechanism for the automatization of movement sequences is the merging of isolated movements into chunks that can be executed as units. In turn, chunks can be utilized hierarchically, as building blocks of longer chunks. It is likely that these mechanisms apply also to speech, so that frequent syllables and words are produced as motor chunks. It is further indicated that the main learning principle for sequence learning is reinforcement learning, with the phasic release of dopamine as the primary teaching signal indicating successful sequences. It is proposed that the dynamics of the dopamine system constitute the main neural basis underlying the situational variability of stuttering.

## 1 Introduction

### 1.1 Background and Aim

Stuttering is a speech disorder, which core symptoms manifest as an intermittent loss of volitional control of the speech movements, resulting in various forms of speech disruptions and speech motor abnormalities (Perkins, 1990; Bloodstein and Bernstein-Ratner, 2008). This means that stuttering is displayed as a disorder of motor execution. Research on stuttering has made great progress in recent decades, but our understanding of the fundamental nature of stuttering is still fragmentary, at best. The dopamine system has been suggested to be implicated in stuttering, in particular because of pharmacological effects and because of theoretical links between stuttering and the basal ganglia (Wu et al., 1997; Maguire et al., 2002; Alm, 2004; Chang and Guenther, 2020; Jenson et al., 2020).

The present review on dopamine and motor automatization was written as part of a research program on stuttering and speech, within a series of theoretical articles. The motivation came from indications that the dopamine system and the neural mechanisms for automatization are likely to be fundamental for the childhood acquisition of speech, and are likely to also be involved in the mechanisms of stuttering in some ways. The research on the cerebral dopamine system has progressed rapidly, and novel methods such as optogenetics provide new information about the differing roles of individual dopamine neurons. A central problem in the research on dopamine has been to reconcile its many different functions, related to reward, motor control, learning, etc. The aim of this article was to provide a brief general overview of the current understanding of the cerebral dopamine system, and more specifically to focus on mechanisms related to the automatization of motor sequences, which may be of importance for the understanding of speech and stuttering. It should be emphasized that this article was not primarily intended to be a review of the existing research on speech or stuttering in relation to dopamine or automatization, but rather to present a physiological framework for further research on these topics. However, speech and stuttering are discussed briefly in appropriate contexts, and some possible implications of the reviewed research are included.

The functions of the cerebral dopamine system are to a large extent linked to the basal ganglia, and it is assumed that the reader has some familiarity with the anatomy and functions of these structures. For a summary of “classical” models of the basal ganglia I refer the reader to section 2 in Alm (2004), which provides a review and discussion of possible links between stuttering and the basal ganglia system.

### 1.2 Automatization of Motor Sequences and Speech

Automatization of motor behaviors implies that a sequence of separate movements becomes well learned, and may be executed with little or no attention. In our everyday life automatization is of great importance, allowing us to, for example, shift gears in a car while remaining attentive to traffic. Despite motor automatization being a fundamental function of the motor system, it has been difficult to pinpoint how it is learned in the brain, or how automatized sequences are executed.

Speech is one of the most complex motor behaviors in humans, as well as one of the most automatized. This automatization usually makes it possible to produce well-articulated rapid movement sequences, without conscious attention to the actual movements. This ability is typically learned and automatized during childhood. Our understanding of the neural underpinnings of speech has increased over the last decades (for a comprehensive overview, see Guenther, 2016). However, our understanding of the mechanisms underlying the automatization of speech motor production remains limited.

In relation to stuttering a few studies focusing automatization of motor sequences exists, with mixed results. The possible links between stuttering and automatization were reviewed by Smits-Bandstra and De Nil (2007). They concluded that adults who stutter tend to show deficits in the learning of finger tapping and nonsense syllable sequencing. This was supported by a later study of the learning of non-words in adults (Namasivayam and van Lieshout, 2008). However, these results were contradicted by two recent studies of finger sequence learning, in adults (Korzeczek et al., 2020) and children (Tendera et al., 2020), reporting no group difference in sequence learning. Instead, the latter study found indications of more general fine motor difficulties. Further, “implicit sequence learning” refers to learning of sequences without the ability to verbally describe the sequence. In two studies of implicit syllable sequence learning, the learning pattern of the stuttering group was more similar to a group of patients with Parkinson’s disease than to the typical control group (Smits-Bandstra and Gracco, 2013, 2015). This was interpreted as indicating possible dysfunction of the basal ganglia loops.

It should be emphasized that the basal ganglia are parts of an extensive network that includes the cerebral cortex, the thalamus, and not least, the white matter connections. Symptoms of basal ganglia dysfunction may appear as a result of impairments in other parts of the network. For example, it might be conceived that impaired input to the basal ganglia can make the system unstable and therefore more vulnerable to normal variations in dopamine release.

### 1.3 Organization of the Article

This review consists of two main parts: an overview of the cerebral dopamine system and an overview of the automatization of movement sequences. These two topics are intended to be general, not specifically related to stuttering or speech. The two sections are further addressed in the “Discussion” Section, which is followed by a brief discussion of the symptoms of stuttering in relation to the reviewed information.

## 2 The Cerebral Dopamine System: An Overview

### 2.1 An Evolutionary Perspective on Dopamine and the Basal Ganglia

#### 2.1.1 Conserved Architecture in Vertebrates

In principle, all motile animals actively move to approach resources they need, and move to avoid harmful situations. In animal behavior research, these fundamental behaviors are often used as indicators of rewarding versus punishing properties of stimuli. Rewards can also be described as reinforcers, because they stimulate the learning of the actions that led to the reward (Barron et al., 2010). In vertebrates, the basal ganglia and the neurotransmitter dopamine play central roles in these mechanisms of reinforcement learning of movements (Grillner et al., 2013). The lamprey is a jawless fish, which diverged from other vertebrates about 560 million years ago. Strikingly, in the last decade, Grillner et al. (2013) and Grillner and Robertson (2016) found that the structure and function of the basal ganglia are surprisingly similar in lampreys and other vertebrates. This indicates that the basic principles of the basal ganglia circuits and neurotransmitter systems evolved in early vertebrates, and have been conserved for more than half a billion years. As one example, the distinction between a direct and an indirect pathway, expressing dopamine D_1_ and D_2_ receptors respectively, is shown in lampreys as well as humans. This suggests that the fundamental architecture of the basal ganglia is central for the functioning of the vertebrate brain.

#### 2.1.2 The Basal Ganglia Originally Controlled the Brain Stem

In early vertebrates, the behavioral repertoire was dominated by movement patterns organized by the brainstem and spinal cord, such as locomotion, eye movements, posture, sexual behavior, and defense behaviors. The original function of the basal ganglia appears to have been to control the activation of these behavioral programs, through output to the brainstem: (1) by providing a basic tonic inhibition of motor activity at rest, via the indirect pathway, and (2) by activating specific motor patterns, via the direct pathway (Grillner and Robertson, 2016). In addition to this system mammals developed the neocortex, with a motor system that allows more detailed control of movements (Karten, 2015; Kawai et al., 2015; Kaas, 2019). In the literature on the basal ganglia, in particular in primates, most interest has focused on the loops connecting the cortex and the basal ganglia, as described by Alexander et al. (1986). However, it has been suggested that the importance of downstream output from the basal ganglia to brainstem motor centers has been underestimated in humans and other primates (Grillner and Robertson, 2016). In particular, it has been suggested that some motor symptoms of basal ganglia dysfunction can be related to dysfunctional downstream output to the brainstem (Takakusaki et al., 2004).

#### 2.1.3 The *FOXP2* Gene: Effects on Dopamine and the Basal Ganglia

The *FOXP2* gene became renowned as the first gene discovered to be associated with speech and language and has been called “the language gene,” though “the speech gene” seems more appropriate. Humans with only one functional *FOXP2* gene show impairments of speech motor performance, particularly the ability to produce or imitate multisyllabic sequences. The deficit appears to be related to a reduced ability to produce rapid movement sequences (Vargha-Khadem et al., 2005). The evolution of the human *FOXP2* gene is of great interest, because it shows only one difference (mutation) between mice and chimpanzees but two differences between chimpanzees and humans (Enard et al., 2002). This suggests that the *FOXP2* gene has been of importance for the evolution of specific human skills.

It has later been reported that the humanized *FOXP2* in particular affects the basal ganglia (Enard et al., 2009; Enard, 2011; Reimers-Kipping et al., 2011). Studies have found that the human version of the *FOXP2* gene affects the concentration of dopamine but not that of serotonin, GABA, or glutamate, and that it results in increased dendrite length and increased synaptic plasticity in the striatum. In song-learning birds, periods of vocal learning appear to be associated with elevated expression of *FoxP2* in Area X, in the anterior striatum (Haesler, 2004). In relation to stuttering this is of interest, as specific damage to Area X in adult zebra finches has been shown to result in stuttering-like syllable repetitions in their song (Kubikova et al., 2015). The possible relevance of these findings for human speech disorders is strikingly increased by the finding of convergent genetic evolution of brain regions involved in vocal learning, in song-learning birds and humans (Pfenning et al., 2014).

In conclusion, the results suggest that the mutations resulting in the human version of FOXP2 were important to allow the development of rapid articulated speech. Further, the results suggest that a crucial factor was the ability to learn and execute rapid movement sequences, and that this involved changes within the basal ganglia and the dopamine system.

### 2.2 Basic Anatomy and Physiology

#### 2.2.1 Dopamine Sources in the Brain

**FIGURE 1:**
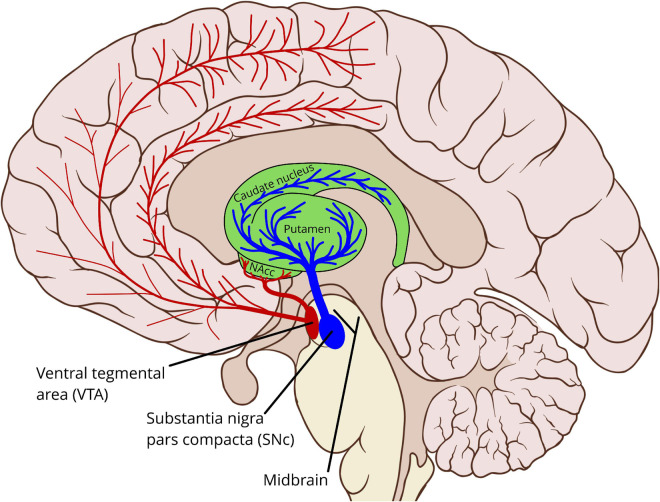
Schematic outline showing the midbrain dopaminergic sources and main pathways to the striatum and the cerebral cortex. In particular the cortical pathways are incompletely characterized, and motoric cortical regions may be innervated by both the VTA and the SNc (Gaspar et al., 1989; Björklund and Dunnett, 2007; Hosp et al., 2019). VTA, ventral tegmental area; SNc, substantia nigra pars compacta; NAcc, nucleus accumbens (The background sagittal brain view is from Patrick J. Lynch, medical illustrator, CC BY 2.5 <https://creativecommons.org/licenses/by/2.5>, via Wikimedia Commons, https://commons.wikimedia.org/wiki/File:Brain_human_sagittal_section.svg. Illustration of the basal ganglia and the dopamine pathways by Per A. Alm).

The brain stem and adjacent regions contain a network of interconnected nuclei, sometimes termed the reticular formation (Ferrucci et al., 2019), which produce the neuromodulators dopamine, serotonin, norepinephrine, acetylcholine, and histamine (van den Brink et al., 2019). These nuclei project to most parts of the cerebrum, including the cerebral cortex and the basal ganglia. Dopamine is primarily produced by two of these nuclei, in the midbrain: the *substantia nigra pars compacta* (SNc) and the *ventral tegmental area* (VTA), see Figure 1. In addition to these midbrain sources, dopamine is also produced by neurons in the hypothalamus, projecting to the pituitary gland via the *tuberoinfundibular pathway.* Dopamine in the pituitary gland inhibits the secretion of prolactin, which is involved in the hormonal system (Fitzgerald and Dinan, 2008).

#### 2.2.2 The Striatum: A Major Target for Dopamine

The basal ganglia consist of a set of gray matter structures, the largest of which is *the striatum*. The striatum receives inputs from most parts of the cerebral cortex, and projects, indirectly, to the frontal lobe and to brain stem nuclei (Coddington and Dudman, 2019; Klaus et al., 2019). These basal ganglia loops can modulate frontal cortical activity and play a fundamental role in motivation, attention, the automatization of behaviors, and the initiation of movements.

The striatum can be divided into a dorsal part and a smaller ventral part. The dorsal striatum can be subdivided based on its inputs, into *the associative striatum* (the caudate nucleus + the anterior putamen) and *the sensorimotor striatum* (the rest of the putamen). The associative part of the striatum receives non-dopaminergic input from the prefrontal cortex and association areas in the temporal lobes, whereas the sensorimotor part receives inputs from the parietal lobes and the motor cortices (Ashby et al., 2010). The ventral striatum primarily consists of *the nucleus accumbens* (NAcc, for localization and shape, see Lucas-Neto et al., 2013). The NAcc projects strongly to the orbitofrontal cortex and plays a central role in emotional evaluation, reward, and motivation.

#### 2.2.3 Dopamine Projections

The functions of the striatum are strictly dependent on a well-regulated input of dopamine from the midbrain. The dorsal striatum receives the highest density of dopamine fibers in the human brain, from the SNc via the nigrostriatal pathway (Yin et al., 2008). In parallel, the NAcc receives dopamine from the VTA, via the mesolimbic pathway. The VTA also provides dopamine to the cortex, via the mesocortical pathway. According to the “traditional” model there is a clear division of the targets for these pathways. However, further studies have shown this to be an oversimplification (Björklund and Dunnett, 2007). For example, the SNc also has neurons innervating the cortex and limbic regions, and the VTA has neurons projecting to the caudate nucleus (Björklund and Dunnett, 2007). The limbic areas receiving dopamine projections include the amygdala and the hippocampus. Dopamine release in these structures is assumed to facilitate memory formation (Hosp et al., 2019).

There seems to be a widespread misconception, that the prefrontal cortex is the primary cortical target of dopamine projections in humans. On the contrary, the highest density of cortical dopaminergic fibers in humans has been reported in the primary and secondary motor regions and in the anterior cingulate cortex (ACC), with lower fiber density in prefrontal regions (Gaspar et al., 1989). Using tractography, Hosp et al. (2019) reported a pathway from the VTA reaching the sensorimotor cortex, the supplementary motor area (SMA), and the dorsal premotor cortex. It has been shown that the presence of dopamine in the motor cortex is necessary for synaptic plasticity and motor learning (Molina-Luna et al., 2009; Hosp and Luft, 2013). In conclusion, both the VTA and the SNc project to subcortical and cortical targets, with substantial overlap.

#### 2.2.4 Dopamine Receptors

Essentially all physiological effects of dopamine are mediated by five subtypes of dopamine receptors: D_1_, D_2_, D_3_, D_4_, and D_5_ (Beaulieu et al., 2015)^1^. The two dominant subtypes of dopamine receptors are the D_1_ and D_2_ receptors, at a level of 10–100 times the number of the other receptors (Hurley and Jenner, 2006). The subtypes are classified as D_1_-class (D_1_ and D_5_) or D_2_-class (D_2_, D_3_, D_4_) (Beaulieu et al., 2015). Basically, the D1 and D2 receptors have opposite effects on the striatal projection neurons, with the D_1_ receptor being excitatory, increasing the likelihood of firing, whereas the D_2_ receptor has an inhibitory effect, decreasing the likelihood of firing (Keeler et al., 2014). The highest expression of the D_1_ and D_2_ receptors, have been reported in the dorsal striatum, consisting of the caudate nucleus and the putamen (Yin et al., 2008). The striatum contain an order of magnitude more dopamine receptors than any other part of the brain (Keeler et al., 2014). Type D_2_ receptors are expressed at very low levels in the cerebral cortex, whereas the type D_1_ receptors can be found at moderate levels throughout the cortex, with the highest density in the medial frontal parts (Hurd et al., 2001). Both the D_1_ and D_2_ receptors are expressed as postsynaptic receptors, however, the D_2_ receptor also acts as a presynaptic autoreceptor in the striatum, thereby regulating the release of dopamine. This feedback loop implies that drugs targeting the D_2_ receptor may have complex effects, acting both pre- and postsynaptically. There is a small difference in the amino acid sequence of the post- and presynaptic D_2_ receptors, which can result in somewhat different pre- versus postsynaptic affinity for different drugs (Usiello et al., 2000).

#### 2.2.5 Dopamine Release

The midbrain dopamine neurons at rest fire at a low stable rate, of approximately five spikes per second (Dodson et al., 2016) but sometimes show brief bursts of firing for approximately 100 ms (Howe and Dombeck, 2016). More specifically, VTA dopamine neurons can switch between three different states: inactive, active tonic firing 2–4 Hz, and phasic burst firing >15 Hz (Douma and de Kloet, 2020). The stable firing results in a baseline “tonic” level of dopamine in the synaptic cleft, whereas the variations in firing encode various events, for example, the learning of behaviors associated with rewards (Coddington and Dudman, 2019). Normally, high levels of synaptic dopamine in the striatum are quickly removed from the synaptic cleft by the *dopamine active transporter* (DAT), thereby maintaining the tonic level of extracellular dopamine in the striatum (Ferris et al., 2014). The cortex differs from the striatum due to low levels of DAT, resulting in slow removal of dopamine that has been released into the cortical synaptic clefts (Helie et al., 2015). In rats, the delivery of a food pellet or the introduction of a new environment has been shown to increase frontal cortex dopamine by about 50%, for about 30–40 min (Feenstra and Botterblom, 1996). These authors proposed that the level of extracellular dopamine in the frontal cortex may reflect increased arousal, which can be positive or negative (reward or stress). Knockout mice lacking DAT get an elevated tonic extracellular level of dopamine in the striatum, and show spontaneous hyperlocomotion (Giros et al., 1996).

### 2.3 Functions of Dopamine

#### 2.3.1 Dopamine Encoding of Subjective Value and Goals

The critical importance of the dopamine system for a range of brain functions has long been recognized. However, clarifying the exact functions of dopamine has been difficult. A reason for this difficulty is probably that dopamine fulfills several purposes in parallel. The details of dopamine functioning continue to be explored, resulting in continuous development of theoretical implications. Berke (2018) attempted to reconcile the multiple experimental findings, proposing that, on the one hand, rapid, phasic dopamine signaling can serve as a “teaching signal” for learning, and, on the other hand, dopamine can represent motivational value and promote movements. His proposal was that (1) the effects of dopamine vary depending on the target region, and (2) target neurons have the ability to switch between learning and performance modes, allowing them to “interpret” the signal in its context. The basic function in common for these two aspects was proposed to be that dopamine signaling “provides a dynamic estimate of whether it is worth expending a limited internal resource, such as energy, attention, or time” (Berke, 2018, p. 787). The motivational value of an event is primarily encoded by the dopamine signaling from the VTA to the NAcc, whereas movements are mainly controlled by the dopamine signaling from the SNc to the putamen and the caudate nucleus.

#### 2.3.2 Dopamine and Initiation of Movement

It has been shown that the dopamine signal from the VTA is closely related to the force (or vigor) of motivated movements (Hughes et al., 2020). The dopamine signals of the SNc primarily encode *if* and *when* a planned movement should be initiated (Howe and Dombeck, 2016; Klaus et al., 2019). However, the dopamine release from the SNc dopamine also influences the vigor of the movement (da Silva et al., 2018). Different subpopulations of dopamine neurons show different patterns of variation in relation to initiation of movement (Klaus et al., 2019). Whereas some neurons show a rapid burst before the onset of a movement (Howe and Dombeck, 2016; da Silva et al., 2018), others show a brief *pause* immediately after the onset of the movement (Dodson et al., 2016).

#### 2.3.3 Specificity of Dopamine Neurons

The dopamine signal from the SNc has been assumed to be non-specific and to generally promote actions that have been planned elsewhere. However, more recent data from optogenetics (Jin and Costa, 2015) indicate that individual dopamine neurons in the SNc may be associated with specific movement sequences.

#### 2.3.4 Movement Preparation

Before a self-initiated movement occurs, a gradual increase in firing can be observed in the dorsal striatum and the motor cortices, which can be detected hundreds of milliseconds to seconds prior to the movement (Romo and Schultz, 1992; Schultz and Romo, 1992). Klaus et al. (2019) proposed that this firing indicates that premovement neural activity can reverberate in the cortico-basal ganglia thalamocortical loops until the movement is activated.

#### 2.3.5 Input Regulating Dopamine Release

An important question is, what inputs cause the SNc to generate a burst of firing to promote movement? Beeler and Dreyer (2019) argued that the midbrain dopamine system and the basal ganglia are core parts of an “axis of agency” that initiate motivated behaviors. They proposed that phasic dopamine signaling occurs when the convergent inputs from diverse regions of the brain show sufficient synchrony and “consensus.” Relatively recently the input from the habenula have become emphasized as a particularly important regulator of dopamine release (Namboodiri et al., 2016).

#### 2.3.6 Dopamine Release During Stress and Aversive Stimuli

As discussed in Section 2.3.1, the release of dopamine has typically been associated with an estimation of subjective value and goal-directed behavior. However, a complicating factor is that stress and aversive stimuli, such as pain, also result in release of dopamine from the VTA to the NAcc and the cortex. How can these observations be reconciled?^2^

First, an important distinction can be made between active and passive stress coping strategies. Active coping strategies involve some type of action, such as fight or flight. Passive coping strategies involve the inhibition of action; for example, in situations of social defeat. Research has shown that active coping strategies are associated with an *increased* release of dopamine in the NAcc, while passive strategies are associated with a *decreased* release (Douma and de Kloet, 2020). In general, aversive stimuli that can be escaped tend to result in an increased level of dopamine in the NAcc, while inescapable aversive stimuli tend to result in a decrease in dopamine, and passivity. These mechanisms are implicated in stress-induced depression, and chronic severe stress may result in degeneration of VTA dopamine neurons (Fox and Lobo, 2019; Douma and de Kloet, 2020). Overall, exposure to chronic stress tends to result in decreased levels of dopamine in the NAcc.

Actions that result in escaping from aversive stimuli are highly rewarding, so in this way both aversive and appetitive stimuli stimulate action, as long as the goals are perceived as attainable. This means that phasic dopamine release can serve as a teaching signal for learning actions in both positive and negative contexts (Stelly et al., 2019).

Another complicating factor is that there are two subpopulations of VTA dopamine neurons, which involve opposite responses to acute stress. Most neurons in the dorsolateral VTA show reactions that are consistent with dopamine as an estimate of value, with inhibition by acute stress and phasic release of dopamine upon *termination* of the stressor. However, dopamine neurons in the ventromedial VTA show strong phasic firing at the *onset* of stressor exposure (Douma and de Kloet, 2020). These two subpopulations appear to have different targets, as de Jong et al. (2019) found that dopamine terminals in the medial shell were excited by aversive events, whereas dopamine terminals in other regions of the NAcc were inhibited by these events.

### 2.4 Dopamine Neurons Have High Energy Demands

Bolam and Pissadaki argued that the dopamine neurons of the SNc are unique in terms of their number of synapses and energy demands (Bolam and Pissadaki, 2012; Pissadaki and Bolam, 2013). They estimated that a single SNc dopamine neuron gives rise to between 1 million and 2.4 million synapses, and has a total axonal length of about 4.5 m (Bolam and Pissadaki, 2012). In addition, the axons are unmyelinated, which further increases the energy demands. In humans, the number of synapses per SNc dopamine neuron is estimated to be about 10-fold higher compared with rats (Bolam and Pissadaki, 2012).

The VTA dopamine neurons appear to have a lower number of synapses compared with SNc neurons. For rats, it was estimated that the VTA neurons provide approximately one-tenth of the number of striatal synapses compared with the SNc neurons, though this estimate did not include the VTA projection to the cortex and other structures outside the striatum (Bolam and Pissadaki, 2012). Another difference between SNc and VTA dopamine neurons is that the DAT is expressed at lower levels by the VTA neurons. The DAT can be a pathway for toxins to enter dopamine neurons, which makes SNc neurons more susceptible compared with VTA neurons.

Bolam and Pissadaki (2012) emphasized that the unique structure of SNc dopamine neurons results in an energy demand that is orders of magnitude larger than that for other types of neurons. In addition, they proposed that most biological functions of the dopamine neurons are under higher demands because of this architecture, such as protein synthesis, cytoskeleton maintenance, and axonal transport. Under normal circumstances these high demands would have no negative effects on the neurons; though, Bolam and Pissadaki argued that these neurons are operating with small margins, so they may be particularly vulnerable to metabolic disturbances, such as mitochondrial dysfunction or oxidative stress. There are, however, indications that the number of synapses per neuron might not directly affect the vulnerability of the neurons: it has been proposed that energy is supplied in the direct vicinity of the synapses, by astrocytes (Calì et al., 2019).

## 3 Movement and Automatization

### 3.1 What Is Automaticity?

Novel motor tasks that are performed with conscious attention activate a large cortical network (Schneider, 2009). Training normally makes the task more automatic, and at a later stage, trained tasks may be performed with little or no attention. An automatized behavior can be produced with greater skill and speed, while requiring substantially reduced neuronal signaling and energy compared with consciously attended behaviors (Schneider, 2009).

### 3.2 Merging Movements Into Action Sequences: Chunking

**FIGURE 2:**
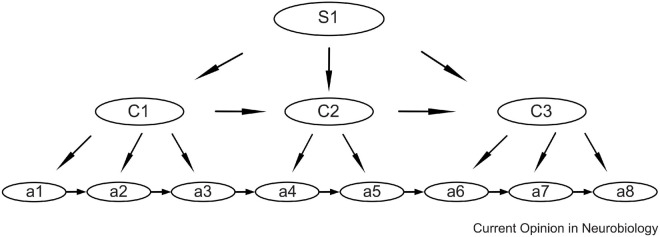
Schematic showing the hierarchical organization model of action sequences, with sub-sequences as sub-chunks. For speech the lowest levels (a) may be the movements of individual speech muscles, the next level (C) may be phonemes, and the higher level (S) may be syllables. Syllables may, in turn, be chunked into automatized multisyllable words. Reprinted from Jin and Costa (2015) with permission.

To optimize performance and reduce neural load, the brain has the ability to combine isolated movements into automatized action sequences or “chunks” (Sakai et al., 2004; Graybiel and Grafton, 2015). These basic chunks can then be organized hierarchically, as shown in Figure 2 (Jin et al., 2014; Jin and Costa, 2015). This means that short motor sequences, or gestures, may be used as building blocks for the automatization of longer sequences.

### 3.3 Learning Principles

#### 3.3.1 Learning of Sequences Based on Feedback of Outcomes

Learning generally occurs through modifications of the synaptic network, such as strengthening or weakening of synapses, or pruning (Pennartz, 1997), which also can be described as neuroplasticity. Which learning principles control this plasticity? First, behaviors that are automatized need to be useful. Instrumental actions have a goal, an intended result. The result of an action should be evaluated in terms of the reward value of the outcome and the cost of the action (Graybiel and Grafton, 2015). “Cost” can be defined broadly, as the amount of energy, time, attention, pain, or risk associated with a given action. To learn and automatize actions, the process must be guided by some type of feedback, allowing only those actions that approach the goal to be reinforced and learned, while actions not approaching the goal are not learned. This principle has been termed *reinforcement learning* (Dezfouli and Balleine, 2012; Robbins and Costa, 2017; Boraud et al., 2018). It has been argued that a core function of the dopamine system and the basal ganglia is to support reinforcement learning in the brain, by providing evaluation feedback (Ashby et al., 2010; Graybiel and Grafton, 2015; Helie et al., 2015; Caligiore et al., 2017). The dopaminergic nuclei in the midbrain receive input from diverse regions of the brain, which can provide the necessary feedback for the appropriate learning of actions.

#### 3.3.2 Differing Learning Principles

**FIGURE 3:**
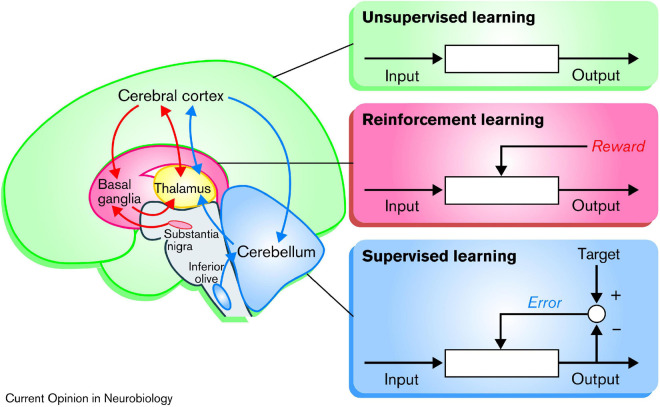
Schematic illustration showing the three different types of learning: According to this model, the basal ganglia are specialized for reinforcement learning, based on rewards in the form of dopamine signaling. The cerebellum is specialized for “supervised learning,” which adjusts the output based on movement error, independent of reward. Finally, the cerebral cortex is specialized for unsupervised “Hebbian” learning, which, might be modulated by inputs from the basal ganglia. Reprinted from Doya (2000) with permission.

It has been proposed that the basal ganglia, the cerebellum, and the cerebral cortex are specialized for three different principles of learning, respectively (Doya, 2000; Caligiore et al., 2017), as illustrated in Figure 3: (1) As discussed in the preceding paragraph, the basal ganglia appears to be specialized for *reinforcement learning*, based on the dopamine teaching signals from the VTA and SNc. (2) In contrast, the cerebellum appears to be associated with *error-based learning*, also known as *supervised learning*, which occur independently from reward, based on mechanistic minimization of movement errors relative to the intended target. (3) Lastly, the cortex has been proposed to be specialized in *unsupervised* or *Hebbian learning*, based on the principle that “neurons wire together if they fire together” (Lowel and Singer, 1992, p. 211). This principle can be described as “blind learning,” because any behavior that is repeated will be strengthened. Though, it has also been suggested that the subcortical input originating from the basal ganglia can act as teaching-signals for this Hebbian learning (Ashby et al., 2010; Caligiore et al., 2017).

### 3.4 Brain Structures and Motor Automatization

#### 3.4.1 The Sensorimotor Striatum (the Putamen) and Dopamine Receptors

Despite the central importance of motor automatization, the exact underlying neural processes remain matters of debate. The primary neural components appear to involve the basal ganglia (including dopamine signaling), the cerebellum, the preSMA, and the primary motor cortex. Overall, one can expect a transition of activity from executive and associative regions of the brain to sensorimotor regions during automatization of movements. In the striatum, this is reflected as a stronger involvement of the caudate nucleus in early learning, and a transition to the putamen in later phases of motor learning (Ashby et al., 2010; Durieux et al., 2012).

As discussed in Section 3.2, above, an important process in automatization of movements is chunking, to allow the sequence to be initiated as a single unit. In the striatum, prominent firing of striatal projection neurons can be observed at the beginning and the end of an automatized chunk, with reduced activity in between. For some striatal neurons, the firing that occurs during a chunk is even lower than that during baseline rest. This firing pattern can be exemplified by observations made with animals running in mazes. During the initial training period, the striatal projection neurons fires during the entire run. However, as learning increases, the activity becomes more prominent at the beginning and the end of the run and declines during the period in between (Graybiel and Grafton, 2015).

**FIGURE 4:**
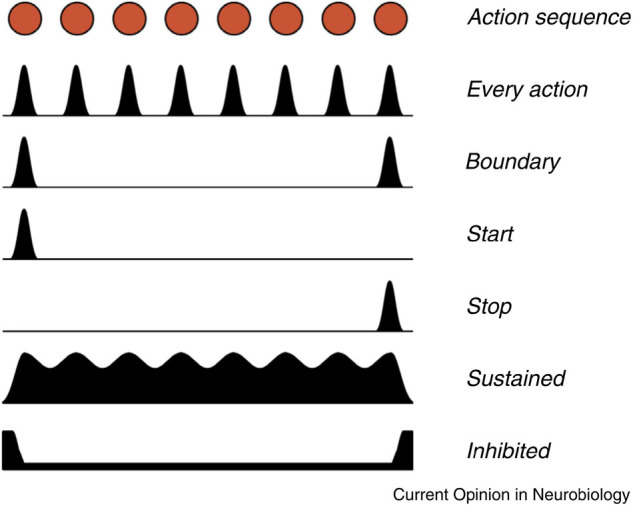
Different patterns of signaling were observed in neurons in the basal ganglia during the execution of a learned action sequence with eight units. Based on recordings from the SNc, striatal projection neurons, and from basal ganglia output neurons, in mice. The red dots at the top represents a timeline of eight actions in an action sequence. The black surfaces represent the variations in firing in different populations of basal ganglia neurons. For example, one population signals for every action, while other neurons only signal at the start and the end of the sequence. Reprinted from Jin and Costa (2015) with permission.

Jin and Costa (2015) summarized animal studies of basal ganglia activity associated with action sequences. Figure 4 illustrates different patterns of basal ganglia firing during the execution of a learned action sequence. The basal ganglia neurons show diverse patterns, indicating specific functions, with emphasis on the beginning and the end of the sequence. In the SNc there are individual neurons firing either for every action (e.g., lever pressing), for the start of the sequence or for the stop. Similar patterns can also be observed in the striatum. In addition, individual dopamine neurons in the SNc appear to be specifically associated with certain sequences. These findings clearly indicate that dopamine signaling in the SNc is not simply a collective on/off process but is much more subtle, possibly related to the somatotopic organization of the putamen.

The results of optogenetic studies reported by Tecuapetla et al. (2016) suggest that the direct and indirect pathways from the striatum play complementary roles in the initiation and execution of action sequences. The established model is that activation of the direct pathway disinhibits motor behaviors, while the indirect pathway suppresses motor activity (Grillner and Robertson, 2016). The neurons in the direct pathway express excitatory D_1_ receptors, whereas those in the indirect pathway express inhibitory D_2_ receptors, implying that dopamine release in the putamen would facilitate motor activity through its effects on both pathways. The results from Tecuapetla et al. (2016) support the view that the activation of neurons in the direct pathway is important for the initiation of a sequence, in line with the established model. However, regarding the indirect pathway, a different dynamic was shown: The proper level of activation of neurons in the indirect pathway neurons was required for the learned sequence to continue; both excessive and insufficient firing of the neurons in the indirect pathway resulted in abortion of the ongoing sequence. In consequence, it appears that either too low or too high levels of synaptic dopamine could result in disruption of the execution of learned sequences.

It has been modeled that phasic versus tonic dopamine stimulation has differential effects on the D_1_ and D_2_ receptors, with bursts primarily increasing D_1_ occupancy whereas pauses in firing reduce the occupancy of both D_1_ and D_2_ receptors (Dreyer et al., 2010). This may imply that phasic dopamine release primarily activates D_1_ receptors and the direct pathway, while tonic dopamine release would result in relatively stronger D_2_ activation, in turn inhibiting indirect pathway firing.

#### 3.4.2 Cortical Regions: The Presupplementary Motor Area

The chunking of movements into sequences has also been demonstrated at the level of the preSMA (Sakai et al., 2004). Nakamura et al. (1998) investigated the learning of button press sequences in monkeys. At the beginning of learning, neurons in the preSMA signaled before each action in the sequence. However, as learning progressed, a chunking pattern emerged, starting as very short chunks and gradually developing into longer chunks until the whole sequence was executed as one chunk. The majority of the neurons in the preSMA primarily signaled at the *initiation* of a chunk. In contrast, the neurons of the SMA proper primarily signaled *during* the execution of the learned sequences, and are likely involved in the initiation of individual muscle contractions.

The observation that the preSMA works at the chunk level is supported by a study that used transcranial magnetic stimulation (TMS) in humans learning a 12-movement finger sequence. After extensive training, an individual pattern of chunking occurred, which was manifested as variations in the temporal patterns of the movements. TMS applied to the preSMA affected the execution only when applied between chunks, with no effect when applied during a chunk (Kennerley et al., 2004). In contrast, TMS applied to the lateral premotor cortex did not affect the movement execution, regardless of when it was applied (Interestingly, TMS over the preSMA before the start of the sequence only affected the initiation of the sequence if no sensory cue was provided for the first movement of the sequence).

#### 3.4.3 The Cerebellum

During movements, the cerebellum has a real-time modulatory effect on the primary motor cortex neurons (Purves et al., 2001, p. 403). According to Stoodley and Schmahmann (2010), the cerebellum can be divided into sensorimotor parts (lobules I–V and lobule VIII), cognitive/associative part (lobules VI–VII), and limbic parts (the posterior vermis). The distinction between novel and automatized movement sequences also appears to be reflected in the cerebellum, with a shift that reflects a transition from cognitive/associative processing to sensorimotor processing (Sakai et al., 2004).

#### 3.4.4 Execution of Learned Motor Sequences Without the Motor Cortex?

In Section 2.1.2, it was suggested that the role of the downstream motor output of the basal ganglia generally is underestimated in humans and other primates (Grillner and Robertson, 2016). It was argued that when the basal ganglia originally developed, they controlled the motor programs of the brainstem and the spinal cord. Recent studies by the Ölveczky Lab have shown that rats trained to perform a sequential task with spatiotemporal precise movements, without demands for dexterity, could perform this task even after removal of the motor cortex, without discernible impairment of performance (Kawai et al., 2015; Dhawale et al., 2019). The task required the rat to press a lever twice with the prescribed timing to get a reward. However, the motor cortex was necessary for the *learning* of the sequence, as rats with lesioned motor cortex before training were unable to learn the required timing. In cats, complete neonatal removal of the cerebral cortex has been reported to result in surprisingly limited deficits, as the cats learned to walk, eat, drink, and groome themselves adequately, guided by both vision and tactile senses (Bjursten et al., 1976). Rhesus monkeys have been shown to recover remarkably after unilateral removal of the sensorimotor cortex, even when the lesion occurs at adult age (Passingham et al., 1983). They are reported to walk, climb, and jump with ease, but are not able to grip food using only the thumb and forefinger. Based on these findings, Kawai et al. (2015) argued that it is clear that the motor cortex is not the only structure that is capable of commandeering motor circuits within the brainstem and spinal cord, and that the subcortical motor infrastructure is quite sophisticated.

In humans it is less clear to which extent the basal ganglia can drive actions independently of the motor cortex. In summary, it seems important that motor research on humans, in particular, research on basal ganglia motor disorders, also consider the possible contributions of downstream motor output from the basal ganglia. It seems likely that dysregulated downstream output from the basal ganglia can interfere with normal motor control in some conditions.

### 3.5 Automatization of Speech Movements

According to the GODIVA model, a computational model of speech sound sequencing, chunking is the basis for automatization of frequent sequences of speech movements, such as syllables (Bohland et al., 2010; Guenther, 2016, p. 237). This is in line with Hickok’s (2014) proposition that frequent syllables and words can be efficiently coded as motor chunks, corresponding to “the mental syllabary” outlined by Levelt and Wheeldon (1994). In a study of training of novel phoneme sequences, by Segawa et al. (2019), it was shown that consonant clusters tend to be learned as units, which generalize to new syllables containing these clusters.

Functional magnetic resonance imaging (fMRI) has also been used to study the automatization of speech. Using fMRI, Alario et al. (2006) found indications that the sequencing of syllables is controlled by the posterior part of the preSMA. This is in line with increasing involvement of the preSMA in more complex syllable sequences (Bohland and Guenther, 2006). Peeva et al. (2010) reported that processes related to phonological chunking are linked to activation of the right superior lateral cerebellum. Further, using fMRI, Segawa et al. (2015) showed that novel sequences of phonemes resulted in higher activation of a network of cortical regions such as the preSMA, the lateral premotor cortex, the ventral primary motor cortex, and auditory regions, together with the basal ganglia.

In conclusion, the findings and theoretical constructs regarding automatization of speech indicates that it follows the general principles of motor automatization, as reviewed in previous sections.

### 3.6 Freezing of Gait: An Example of Basal Ganglia Dysfunction

Freezing of gait (FoG) is a symptom of Parkinson’s disease, with unclear pathophysiology (Chen et al., 2019). FoG is of particular interest in the context of the present article, as it affects the execution of a highly automatized behavior, walking, and it shares several characteristics with stuttering. The following description of clinical features of FoG is based on the review by Nutt et al. (2011): (1) FoG appears as brief periods of inability to move the feet forward, despite the intention to walk (or marked reduction of the amplitude of the movements). (2) FoG typically occurs when starting to walk, or when the person needs to change the walking pattern (e.g., when turning). In the latter case, it leads to the arrest of the ongoing movement. (3) Commonly such episodes of FoG last a couple of seconds, but may exceed 30 s. In rare cases the symptoms are continuous. (4) Leg tremor at a frequency of 3—8 Hz often occurs during FoG. (5) Episodes of FoG are accompanied by a subjective feeling of the feet being glued to the floor. (6) FoG is commonly relieved by various sensory cues, for example a rhythm to follow. (7) The emotional and cognitive situation, with environmental influences, can have striking effects on FoG.

Regarding the level of muscular tension, electromyographic investigation has shown that the tension of the leg muscles is not elevated, or characterized by co-contraction (Nieuwboer et al., 2004). Nutt et al. (2011) described FoG as a mysterious phenomenon. A recent clue to the pathophysiology comes from a study by Chen et al. (2019), associating elevation of certain oscillatory frequencies in the basal ganglia with risk for FoG.

## 4 Discussion

The anatomy and physiology of the dopamine system have been reviewed above, together with a review of mechanisms for automatization of motor sequences. These two topics will be summarized and discussed below, to be followed by a discussion of the symptoms of stuttering in relation to the reviewed topics.

### 4.1 The Dopamine System

In most instances, and in most regions of the brain, the release of dopamine tends to signal subjective value, motivation, and action, in the context of both approaching an appetitive stimulus and avoiding an aversive stimulus (Berke, 2018). It appears that the perceived attainability is of key importance for the release of dopamine and the behavioral response, with an inhibition of dopamine and action if the aversive stimulus is perceived to be unavoidable (Douma and de Kloet, 2020), or if the appetitive stimulus is perceived to be unattainable. In this sense, the motivation for action would be based on a combination of subjective value and the perceived attainability.

A minority of dopamine neurons show “atypical” responses, with burst firing at the onset of aversive events. They are located in the ventromedial VTA and appear to project to the medial shell of the NAcc (de Jong et al., 2019; Douma and de Kloet, 2020). It is possible that the function of these neurons is to increase the level of attention and arousal in moments of perceived danger, whereas dopamine neurons with typical pattern of signaling are more related to approaching and evaluating the possible outcomes of actions.

The review indicates that the dopamine system can be viewed as a core component in basically all human behavior, conveying a compound estimate of subjective evaluations, as well playing a central role in both the learning and execution of automatized action sequences. It is clear from the present review that this system may show functional problems in a multitude of ways, with differences apparent in symptomatology and pharmacological responses.

A key aspect that may be of relevance for various pathologies affecting the dopamine system is the extreme architecture and high energy demands of dopamine neurons, in particular the SNc neurons. This has been proposed to make these neurons vulnerable to relatively minor disturbances of the metabolism (Bolam and Pissadaki, 2012).

### 4.2 Movements and Automatization

#### 4.2.1 Automatization of Sequences

In summary, a primary mechanism for automatization of movements is the merging of isolated movement into “chunks” that can be executed as a unit (Jin and Costa, 2015), with little attention. In turn, chunks can be utilized hierarchically, as building blocks in longer chunks, as illustrated in Figure 2. The results from animals indicate that some neurons in the SNc and in the striatum signals for each submovement in a chunk (Jin and Costa, 2015). The principle of chunking appears to also be involved in the automatization of speech (Bohland et al., 2010; Guenther, 2016, p. 237). The normal learning of sequences tends to be based on reinforcement learning, with phasic release of dopamine as the primary teaching signal indicating successful sequences.

The review suggests the existence of two parallel networks for the execution of learned sequences: (1) one network for the start of sequences, involving the basal ganglia and the preSMA and relying on the activation of the D_1_ receptors of striatal neurons forming the direct pathway, (2) one network for the continued execution of learned sequences, involving the sensorimotor parts of the basal ganglia, the cerebellum, and the SMA proper. The continued execution relies on a balanced activation of the D_2_ receptors of striatal neurons forming the indirect pathway.

#### 4.2.2 Dopamine Signaling for Initiation of Movement

The volitional initiation of movements is dependent on the signaling both from the SNc and the VTA. The data suggest that the SNc provides a fine-grained signal, in which individual dopamine neurons can be linked to specific actions (Jin and Costa, 2015). The signal from the VTA affects the force and vigor of the movement (Hughes et al., 2020). Reverberation of firing in the cortico-basal ganglia thalamocortical loops is likely to be important for the preparation before voluntary movements (Klaus et al., 2019), and will be sensitive to variations in the release of dopamine.

#### 4.2.3 Cues for Initiation of Movement

It is known that auditory and visual cues can facilitate walking in Parkinson’s disease and the fluency of speech in stuttering (Brady, 1969; Suteerawattananon et al., 2004; Bloodstein and Bernstein-Ratner, 2008). The “classical” explanation of this phenomenon is that externally cued movements are initiated by the lateral premotor cortex together with the cerebellum, thereby bypassing the basal ganglia and the SMA (Cunnington et al., 1996; Hanakawa et al., 1999; Haslinger et al., 2001; Wu and Hallett, 2005). As discussed in Section 2.3.5, it has been proposed that the SNc initiates movements when the inputs from diverse regions show sufficient synchrony and “consensus” (Beeler and Dreyer, 2019). This model suggests a somewhat modified mechanism underlying externally cued movements in Parkinson’s disease and stuttering: that sensory cues together with focused attention results in increased synchrony of firing in the sensorimotor system, which can be sufficient to result in dopaminergic firing from the SNc and, in turn, initiation of movement. This model is supported by results showing that auditory signals, in particular rhythms, provide a synchronizing effect for neural activity (Mathias et al., 2020), and that focused attention implies increased synchronization of the neural activity in the involved networks (Womelsdorf and Fries, 2007). The lateral premotor cortex and the cerebellum could still be essential for the effect of external cues, by extracting the relevant information from the stimuli (Penhune et al., 1998; Hanakawa et al., 1999).

It should be emphasized, however, that such similarity between stuttering and Parkinson’s disease would not in itself implicate that stuttering is related to a similar dopaminergic pathology as Parkinson’s disease. For example, it is likely that other forms of dysregulation of this system also can result in insufficient initiation of speech movements but improved function with the support of external cues.

#### 4.2.4 Paradoxical Movements in Emotional States

Another phenomenon in Parkinson’s disease is the occurrence of “paradoxical movements” in relation to emotional states, signifying an unexpected ability to move during situations involving strong emotions, such as fear or anger (Glickstein and Stein, 1991). Similarly, it has been reported that people who stutter tend to speak well under conditions of strong emotions, including fear, excitement, and motivation (Bloodstein and Bernstein-Ratner, 2008, p. 270). Based on experiments it has been claimed that this effect in Parkinson’s disease reflects a general property of the motor system, of greater vigor during urgency (Ballanger et al., 2006; Thobois et al., 2007). A possible underlying mechanism is that emotional urgency results in increased synchrony of the inputs to the VTA and SNc, with stronger dopamine release increasing the force and vigor of the movement, as described by da Silva et al. (2018) and Hughes et al. (2020).

#### 4.2.5 Execution of Sequences Without the Motor Cortex

Recent studies by the Ölveczky Lab show that rats can perform learned motor sequences with high temporal demands without the motor cortex. This finding carries the important implication that different types of motor sequences are learned and executed in different ways. Sequences involving “dexterity” required the motor cortex both for learning and execution, while sequences of simple movements only required the motor cortex for learning. The implications for human motor control, and for speech, remain to be determined.

In this context it is of interest that human brain lesions with aphasia sometimes result in “speech automatisms,” in particular after lesions including the frontal lobe (Code, 2021). Such automatisms typically take the form of a frequent word or series of words, such as “so and so” or “oh boy” (Code, 1994), or specific consonant-vowel syllables, such as/ba, ba/or/da, da/(Code, 2021). According to the study by Brunner et al. (1982), automatisms only occurred in patients with combined lesions of the left striatum and cortex. It has been hypothesized that the right hemisphere plays a special role in the production of automatisms (Code, 1994). An interesting case was reported by Speedie et al. (1993). After a right hemisphere basal ganglia lesion, the propositional speech of the patient was preserved, but he had lost the ability to recite familiar verses. There was an impairment of the production of serial automatic speech, singing, recitation of rhymes, and swearing. His propositional speech no longer included overlearned phrases. One interpretation of these phenomena is that the right basal ganglia normally has the capability to drive the production of frequently used utterances and songs without the cortex. This function may become disinhibited as a result of lesions of the left hemisphere basal ganglia and cortex, and produced as automatisms.

Parallels between automatisms in aphasia and verbal tics in Tourette syndrome have been discussed by Code (1994, 2021). Similar to the automatisms in aphasia, such verbal tics tend to be uttered in a stereotyped manner. It has been proposed that the verbal tics are produced involuntarily by an interaction between the limbic system and the basal ganglia, uninhibited by the cortical system (Code, 1994, 2021). In conclusion, it might be possible that automatisms in aphasia, and verbal tics in Tourette syndrome, are expressions of the (right) basal ganglia driving motor actions via downstream output to the brain stem.

### 4.3 The Symptoms of Stuttering

#### 4.3.1 Stuttering in Relation to the Degree of Automatization of Speech

Anderson (2007) found that stuttering in preschool children tended to occur more often for words with lower frequency of occurrence in the language, or on words with unusual phonological sequences (after controlling for word length and grammatical class). This implies that words with a lower degree of motor automatization were stuttered more frequently. The difference in the frequency of occurrence for multisyllabic words was quite large between fluent and stuttered words, with an effect size of 1.3 standard deviations for part-word repetitions and 1.65 for prolongations. The result suggests that poor automatization of speech motor sequences contribute to stuttering.

However, Bloodstein and Bernstein-Ratner (2008) stated: “Virtually any change that can be made in the way a person normally talks is apt to result in much improved or essentially fluent speech for the majority of stutterers, provided the change does not lose its novelty” (p. 268). For example, to imitate a foreign accent. This suggests that stuttering in particular interferes with speech produced in a habitual mode, i.e., with an attempt to utilize an automatized mode of speech production. In other words, the deautomatization of speech tend to reduce the symptoms of stuttering. How can this observation be reconciled with the finding discussed in the preceding paragraph, of more stuttering on words with lower degree of automatization?

One interpretation of this contradiction is that the risk for stuttering is high when the speaker is attempting to talk in an “automatic mode” but the movement sequences are poorly automatized. When talking in a novel way a higher level of conscious control is applied, partly bypassing the mechanisms for execution of chunks. To summarize, the results would be compatible with a model in which speech can be produced in two contrasting modes: “automatic” and “non-automatic.” Stuttering would primarily be linked to the automatic mode of speech production. In this mode the risk for stuttering is higher on words with poorly automatized motor sequences. This is in line with observations that persons who stutter tend to say overlearned words or phrases fluently, for example when swearing (Bloodstein, 1950; Bloodstein and Bernstein-Ratner, 2008).

In a recent study combining speech motor training of novel phoneme sequences and brain imaging (fMRI), Masapollo et al. (2021) found indications that people who stutter do not differ from typically fluent persons in terms of the ability to learn new speech motor sequences, but show impairments in the execution of the learned sequences. In addition, they observed an association between high level of in-scanner speech disfluencies and low activation of left basal ganglia sites. Moreover, the result of Ingham et al. (2013) suggested that people who stutter may consist of two subgroups with regard to the ability to utilize automatization of speech motor sequences from fluency training. Successful final results of the training were predicted by decrease of the activation of the left putamen, the sensorimotor part of the striatum, as measured from the beginning of the training to the end of the initial phase. Such a decrease might by indicative of successful automatization of the novel speech pattern.

#### 4.3.2 The Situational Variability of Stuttering

Stuttering is characterized by its typical variability of the symptoms within individuals, from situation to situation and from day to day (Bloodstein, 1950; Constantino et al., 2016; Tichenor and Yaruss, 2020). At least, two observations from the review may be of relevance for this variability: First, the review by Jin and Costa (2015) suggests that the initiation of each and every submovement in a motor sequence may be associated with the firing of specific dopamine neurons in the SNc. Second, according to the reasoning of Beeler and Dreyer (2019), dopamine signaling occurs when the convergent input to the SNc, from different parts of the brain, show sufficient synchrony and “consensus.” Thus, the dopamine signaling may be described as intrinsically dynamic and varying, depending on the specific situation and the internal state of the person. It is here suggested that the dynamics of the dopamine signaling from the SNc and the VTA during speech is the main neural basis for the situational variability of stuttering.

#### 4.3.3 Stuttering as a Possible Effect of Basal Ganglia Dysregulation

##### 4.3.3.1 Heterogeneity of Symptoms: Both Hyper and Hypo?

We need to consider the possibility that the symptoms of speech labeled as stuttering in reality represent several different neurological mechanisms. Considering the complexity of the underlying neural system it would not be surprising if the output can be interrupted in several different ways, but with partly similar overt symptoms. This was also the result of a simulation study by Civier et al. (2013), of stuttering as an effect of impairments in the basal ganglia thalamo-cortical circuit, either because of dopaminergic abnormalities or because of white-matter abnormalities.

While it is clear that many instances of stuttering involve elevated levels of muscular tension (e.g., Freeman and Ushijima, 1978; Freeman, 1979), it has also been reported that moments of stuttering can show reduced or normal levels of muscular activity (Smith et al., 1996; Smith, 2015). In addition, stuttering sometimes involves tremor in speech muscles, in the 5 to 15 Hz range (Fibiger, 1971; Denny and Smith, 1992; Smith et al., 1993). As discussed in Section 1.1, one of the main lines of research on stuttering links the symptoms of stuttering to the functions of the basal ganglia (Wu et al., 1997; Maguire et al., 2002; Alm, 2004; Chang and Guenther, 2020; Jenson et al., 2020). It is of interest that basal ganglia motor disorders can be associated with excessive tension, as in dystonia (e.g., Kaji et al., 2018), tremor (Hallett, 2014), as well as the absence of elevated tension or co-contraction, as in freezing of gait (Nieuwboer et al., 2004). In the two sections below, two hypothetical basal ganglia mechanisms are discussed in relation to stuttering. Both of these mechanisms would be expected to primarily result in stuttering without excessive muscular tension (though this might develop as a secondary effect).

##### 4.3.3.2 Neural Oscillations and Freezing of Gait

Relatively recent models have linked symptoms of some movement disorders to disturbances of the oscillatory properties of the basal ganglia circuits, for example freezing of gait (as reviewed in Section 3.6) and tremor (see, e.g., Brittain and Brown, 2014; Chen et al., 2019; Halje et al., 2019). The disturbances of the oscillatory properties can be secondary to dopaminergic dysregulation, as in Parkinson’s disease, but can also have other causes. Oscillatory disturbances of the basal ganglia have been discussed in relation to stuttering (e.g., Etchell et al., 2014; Mersov et al., 2016; Saltuklaroglu et al., 2017; Chang and Guenther, 2020; Jenson et al., 2020). Considering the symptomatology of freezing of gait it may be stated that the seven characteristics summarized in the review in Section 3.6 are also characteristics of stuttering (e.g., see Bloodstein and Bernstein-Ratner, 2008):

1) Brief periods of inability to move forward in a movement sequence.
2) Often occurs at the beginning of the sequence: often before the first sound (a “block”) or within the initial part of the first word (Wingate, 1969; Bloodstein and Bernstein-Ratner, 2008).
3) Episodes commonly last a couple of seconds, but may exceed 30 s.
4) Tremor may be shown (at 5–15 Hz, Smith et al., 1993).
5) Episodes of stuttering are often accompanied by a subjective feeling of being “stuck.” As Charles Van Riper (1992, p. 83) stated: “Not disfluency but gluency is the essence of our disorder, we get stuck when we stutter,” with the novel word “gluency” referring to the feeling of speech effectuators being stuck in glue.
6) Stuttering is commonly relieved by sensory cues, for example speaking to the pace of a metronome.
7) The emotional and cognitive situation, with environmental influences, can have striking effects on stuttering.

In summary, the parallels between stuttering and the characteristics of freezing of gait in Parkinson’s disease appears promising for further studies.

##### 4.3.3.3 Stuttering as Failure to Initiate or to Sustain the Execution of an Automatized Chunk

The results from Tecuapetla et al. (2016), as reviewed in Section 3.4.1, illustrate how the execution of a movement sequence might fail to be initiated, or how it might be terminated after initiation. According to the results of Tecuapetla et al. (2016), activation of the direct pathway, including the D_1_ receptors, is necessary in order to initiate an action sequence. In addition, a continuous balanced firing of the indirect pathway is required for the execution of the sequence to continue. Too low or too high a firing of the indirect pathway resulted in termination of the sequence. The firing of the indirect pathway is regulated by the inhibitory D_2_ receptors. Applied to stuttering, a part-word repetition without muscular tension might occur when a sequence is correctly initiated by the direct pathway, but the indirect pathway is either hypo- or hyperactive, resulting in a termination of the sequence and the motor output. When a sequence is terminated prematurely, there will be no end-signal for the sequence, which might result in a restart of the failed sequence. The overt symptom of this could be a part-word repetition. This scenario is speculative, but may serve as an example of possible neural mechanisms, guided by general research on the physiology of the basal ganglia.

#### 4.3.4 Further Research on the Motor Characteristics of Stuttering

For an understanding of the neuromechanics of motor disorders it is important to analyze the characteristics of the motor abnormality in detail. Among others, Courtney Stromsta initiated this type of study of stuttering, using spectrography and lip electromyography (Stromsta, 1965, 1987; Stromsta and Fibiger, 1980). This has been followed by some later attempts with various methods (e.g., Conture et al., 1977, 1985, 1986; Freeman, 1979; Throneburg and Yairi, 2001). It is here proposed that it will be of importance for the understanding of stuttering to continue this work with modern techniques and within an updated theoretical framework, of course, in parallel with work to understand the underlying neurobiological mechanisms of stuttering.

## 5 Conclusion

The review clearly indicates that the basal ganglia and the dopamine system play central roles for the learning and automatization of motor sequences, and there are no indications that this is not the case also for speech. On the contrary, the specific effects of the humanized version of the FOXP2 gene on the basal ganglia and dopamine levels point toward key roles for the evolution of speech and language (Enard et al., 2009; Enard, 2011). Recent research on dopamine suggests a more complex organization than previously shown. For example, individual dopamine neurons in the SNc can be associated with the initiation of specific movement sequences (Jin and Costa, 2015). Another result of interest is the indication that a balanced level of activation of the indirect pathway is required for the execution of a chunk to continue (Tecuapetla et al., 2016). As the neurons of the indirect pathway express inhibitory D_2_ receptors, the model supports the importance of balanced activation of these receptors.

The central mechanism for automatization of movement sequences is the merging of isolated movements into “chunks,” which can be executed as a unit. In turn, these chunks can be used as building blocks in longer chunks. The primary learning principle for this automatization is reinforcement learning, with phasic release of dopamine as a teaching signal.

A remaining question concerns the role of downstream output from the basal ganglia to the brainstem and spinal cord in humans. For example, can the basal ganglia drive the production of overlearned speech, such as habitual phrases, without the motor cortex?

In relation to stuttering, it was here proposed that the dynamics of dopamine signaling constitutes the main basis for the situational variability of stuttering.

## 6 Author Contributions

The author confirms being the sole contributor of this work and has approved it for publication.

## Conflict of Interest

The author declares that the research was conducted in the absence of any commercial or financial relationships that could be construed as a potential conflict of interest.

## Publisher’s Note

All claims expressed in this article are solely those of the authors and do not necessarily represent those of their affiliated organizations, or those of the publisher, the editors and the reviewers. Any product that may be evaluated in this article, or claim that may be made by its manufacturer, is not guaranteed or endorsed by the publisher.

## Footnotes

1 This review focuses the effects of dopamine within the brain. However, it is important to emphasize that dopamine also serves in a range of essential physiological functions in the body outside the brain.

2 For excellent reviews on stress and dopamine, the readers are referred to Holly and Miczek (2016) and Douma and de Kloet (2020).

## Abbreviations

DAT: dopamine active transporter
FoG: freezing of gait
NAcc: nucleus accumbens
SMA: supplementary motor area
SNc: substantia nigra pars compacta
VTA: ventral tegmental area.
